# How to improve the transferability of a 12-week home-space sedentary behaviour intervention for ethnically diverse older adults: a qualitative study protocol of key stakeholder perspectives

**DOI:** 10.1136/bmjopen-2024-091049

**Published:** 2025-04-17

**Authors:** Naureen Akber Ali Meghani, Joanne Hudson, Gareth Stratton, Jane Mullins

**Affiliations:** 1 Swansea University Faculty of Science and Engineering, Swansea, Wales, UK; 2 Faculty of Medicine, Health and Life Science, Swansea University, Swansea, Wales, UK

**Keywords:** Aging, Aged, Health, QUALITATIVE RESEARCH

## Abstract

**Abstract:**

**Introduction:**

In the UK, the number of ethnically diverse older adults (OA) is growing. These individuals suffer complex health issues that are made worse by socioeconomic status, acculturation experiences and language barriers. Additionally, this varied group is the least active and a highly sedentary subgroup in the general population, which poses serious health concerns. Various interventions have been implemented with OAs to reduce their sedentary behaviour (SB) and enhance their physical activity (PA). However, there is still limited research that implements stakeholders’ perceptions in translating the interventions into real-life settings, particularly for ethnically diverse OAs. Therefore, the current study aims to explore stakeholders’ perceptions of the transferability of a 12-week home space intervention for ethnically diverse sedentary OAs, that is, aimed at reducing their SB and increasing their PA.

**Methods:**

Exploratory qualitative research using in-depth interviews (IDIs) and a purposive sampling technique will be employed to recruit stakeholders. Before conducting the IDIs, the primary researcher (NAAM) will discuss the findings of the 12-week home space intervention study for ethnically diverse OAs to explain the intervention, and then the interview will revolve around the transferability of the intervention to transfer the intervention into real-world practice into the stakeholder contexts. A diverse group of stakeholders from Swansea, Wales, UK, representing a range of roles including health promotion professionals, programme leads, service providers, policymakers and researchers will be included. The qualitative data obtained will be analysed using reflexive thematic analysis.

**Ethics and dissemination:**

Stakeholders will be required to provide written informed consent prior to initiation of the study. Ethical approval for this study has been obtained from the College of Engineering Research Ethics Committee (320249732903), Swansea University. The study’s results will be shared with the scientific community through a peer-reviewed journal publication and with study participants through seminars and workshops.

**STRENGTHS AND LIMITATIONS OF THIS STUDY**

- A well-established reflexive thematic analysis approach will be used to understand in-depth perceptions of the stakeholders about the transferability of the intervention.
- The findings of the study will identify any necessary modifications and/or alterations in the intervention to improve the feasibility of its real-world implementation and scale-up.
- The researcher will recruit diverse stakeholders, representing a range of roles including health promotion professionals, programme leads, service providers, policymakers and researchers to transfer the intervention into real-world practice.
- The interviewer is also the intervention designer, which might result in social desirability bias; however, the researcher will try to minimise bias by keeping a reflective journal and undertaking qualitative research training.

## Introduction

 Physical activity (PA) is important at all stages of life, including childhood, adolescence and old age.^1 2^ Being active and less sedentary has been found to be beneficial in preventing and managing detrimental health impacts among older adults (OAs).^3 4^ Nevertheless, PA tends to decrease progressively with age, with OAs being considered as the least active age group^5 6^ as the majority of OAs^7^ are sedentary and do not achieve recommended levels of PA.^8^ Research has revealed that two-thirds of OAs have an inactive lifestyle^9^ and they spend ≥10 hours/day being sedentary.^10^ This indicates that OAs spend 79% of their waking hours engaged in sedentary behaviours (SB).^10^

Unfortunately, the situation is further worsened for OAs from socially disadvantaged backgrounds, who therefore face a greater burden of physical inactivity.^11^ This burden is exacerbated in ethnic minority populations who often come from socially disadvantaged backgrounds and have inactive lifestyles due to well-established social and health disparities.^12 13^ For instance, black minority ethnic (BME) populations have lower PA levels^13 14^ and experience a significantly greater disease burden than non-BME communities^15^ in the UK. The 2022 report highlights the low PA participation rates in black Asian minority ethnic (groups; 2%), which raises an issue of major concern.^16^ This has potential implications in Wales given that the number of minority ethnic OAs is increasing, with a notable influx of ethnically diverse OAs within urban areas.^17^ In addition, OAs from minority ethnic groups are harder to reach, less likely to participate in PA programmes and more prone to drop out from PA programmes.^18^ Therefore, promoting PA among OAs, and in particular, OAs from diverse ethnic communities, should be a global priority as it enables active ageing and slows disability and disease progression.^19^

The UK government invests in substantial efforts to modify the low PA and high SB trends among OAs. However, existing programmes that are implemented fail to demonstrate effectiveness in enhancing PA levels and minimising SB among OAs from minority groups.^20^ This might be due to the fact that previous studies have not focused on home-based activities but rather target activities outside the home that might present numerous barriers for ethnically diverse OAs, including financial difficulties, social obligations, a lack of confidence, language hurdles, a lack of resources and restrictions based on religion and culture.^20^ There is compelling research that the physical environment within the home space has a substantial effect on children’s activity and sedentary patterns.^21^ Nonetheless, limited research has examined the home’s physical surroundings in connection to OAs’ PA and SB levels.

Further, with the spread of the coronavirus disease-2019 (COVID-19), where governments enacted preventive measures to limit participation in outdoor PA, sports and exercise, this placed an obstacle that hindered OAs in leaving their homes, enhancing their sedentary time and reducing PA, potentially increasing the risk of chronic diseases.^22 23^ Importantly, as a result, many OAs have not, or are not able to, minimise their sedentary time^24^ or return to their pre-COVID-19 pandemic levels of PA and time spent outside their home.^25 26^ Therefore, doing PA at home is recommended as a useful strategy to promote health,^27^,^29^ strengthen immunity^30 31^ increase physical functioning^32^ and increase physical fitness.^33^ Promoting modifications in the home environment may facilitate home-based PA and reductions in SB.^34^ Moreover, it has been noted that inactive and sedentary OAs view high-intensity activity as inappropriate and believe that PA should only result from meaningful activities like housework^35^ and that even a minor increase in activity among sedentary OAs can improve health effects.^36^,^38^ Thus, highlighting the need to integrate feasible and innovative interventions for OAs.^39^ In light of this, our 12-week intervention focused on optimising OAs’ home space to enhance PA and minimise SB using a brief health coaching session, pamphlet, weekly reminder messages and wearable activity tracker (WAT). Although a preliminary, feasibility trial has established that the intervention is acceptable for use with OAs, it is not yet known if the home-based PA and SB intervention is transferable to real-world contexts and can be delivered as part of existing initiatives to promote health in OAs.

An intervention’s transferability is considered to be the degree to which an intervention’s effects in one setting may be observed in another,^40^ which is important for promoting health. Interventions for health promotion integrate actions targeting behaviour change, modifications to the physical and social environment and public policy. They provide people with the necessary resources to regulate and improve their own health.^41^,^43^ These interventions are complex^44^,^46^; complexity is also evident in the interface between the interventions and the environments in which they take place.^41 47^ One of the most important considerations prior to implementing an intervention is consistency of intervention outcome when it is transferred from one setting to another, or from research to real-world settings.^41^ Achieving this can present a challenge in health promotion. Even when an intervention has shown to be effective in one context, in other contexts the intervention itself may still be useful, but it may have different effects from the original intervention.^48^ Therefore, it is crucial that stakeholders are involved in order to maximise the possibility of effective implementation of the intervention in real-world settings.^49^

Stakeholders are persons or groups who are accountable for or impacted by decisions pertaining to health and healthcare,^50^ and their involvement in research that informs these interventions is considered essential by various funding bodies.^51^ Engagement of stakeholders and stakeholder organisations can offer crucial insights regarding the potential role and capacity of organisations in supporting the implementation of the intervention (eg, funding, referring), identifying obstacles and enablers to incorporate the intervention in regular service delivery and suggesting strategies and actions (including recommended modifications/alterations) of the intervention to assure it is transferable to, and sustainable in, real-world practice.^49^ However, researchers rarely carry out this crucial phase, and as a result, there is currently a dearth of evidence that offers stakeholder viewpoints to guide the transferability of intervention.^49^ According to Glasgow and colleagues,^52^ the majority of interventions are created and evaluated successfully; however, many of them ‘get lost in translation’ because they are not implemented in a sustainable manner in the various ‘real life’ situations. To facilitate sustainable improvements in OAs’ PA and SB, stakeholders should play a main role in the execution and maintenance of interventions after the completion of research programmes that have delivered and evaluated these interventions.^53 54^

Investigating transferability is essential in shifting intervention into practice.^41^ In this regard, perceptions of stakeholders are a productive and valuable knowledge source^54^,^56^ as they offer a unique viewpoint on the intervention, which is essential to understand its transferability. In fact, their perceptions of the intervention affect their decision to replicate or transfer it, and, if it is transferred, how they modify it to assure the sustainability and adaptability of the intervention in a real-world setting.^41^ In this regard, the 12-week intervention that was designed by the researcher and implemented on the ethnically diverse OAs will be presented to stakeholders to gain knowledge about their perceptions to address intervention transferability. Therefore, the current study aims to explore stakeholders’ perceptions on the design and delivery of a home-based SB intervention implemented with ethnically diverse sedentary OAs to inform transferability (future implementation and scaling up).

## Methods

### Study design

Exploratory qualitative research using semi-structured interviews and a purposive sampling technique was employed from 30 July 2024 to 30 September 2024. In-depth interviews (IDIs) with stakeholders will be used as a means of collecting data for this research. Before the interview, the primary researcher will explain the intervention that has been assessed in a previous feasibility study^57^ where sedentary OAs were asked to wear an activity tracker to remind them to cut down their sitting time (by standing up and moving more), attended a brief health coaching session and were provided with a pamphlet and weekly reminder messages to decrease their SB and enhance their PA within the home. The current formative research builds on this feasibility study by enabling stakeholder engagement to involve end users’ in planning for the intervention’s transferability and scalability, shown to be significant in fostering intervention uptake.^58^ This will help us to understand the practical issues or possible challenges of delivering this intervention at larger scale within current policy and service contexts. The interview will then focus on perceptions of the transferability of the intervention in the stakeholder context. The consolidated checklist, (online supplemental file 1, Checklist) contains guidelines for qualitative research that will be followed in reporting this study.

Population-Intervention-Environment-Transfer Model of Transferability (PIET-T).

As a theoretical, conceptual model for assessing the transferability of health interventions, the PIET model can help with decision-making on intervention transferability. Four key elements are necessary for this transferability: the population, the intervention, the environment in the primary and target settings and the transference of the intervention.^59^ It focuses on the perspective of the decision-makers who want to improve the health status of the target population (P) and their intention to transfer the intervention (I) from a primary context to the target context. Decision-makers might include various scholars, experts, policymakers, stakeholders and organisational leaders.

The decision-making process might consider the views and the needs of the target population (P) and the coordinating team in the target environment (E) to plan, organise and carry out the transfer (T). The population, the intervention and the environment are assumed to influence each other. The combination of these three constructs determines the outcome. The (baseline) population characteristics in the target setting are used to examine the health issue in order to determine an appropriate intervention.

The decision-maker collects information regarding the evidence offered in a primary context (eg, research design, site and demographic characteristics). Before deciding on the possible transfer of the intervention, the decision-maker must take into account the circumstances of both the primary context and his or her own setting, also referred to as the target context. Therefore, it could be necessary to adapt the intervention to the intended circumstance.

The transfer can take place at a number of levels in a target setting including the individual, organisational, local (community or region), national or even worldwide levels. Both the main and target contexts influence outcome transferability. Information from the primary context is used to guide the transfer’s design. Concurrently, as transferability also depends on the interactions between these three constructs, the target contexts must be considered when planning and carrying out the transfer. Therefore, the transferability of health interventions is affected by the population, the intervention, the environment in the primary and target contexts and the transfer process itself. This suggests that for the results of the target context to be comparable to those of the primary context, the constructs in both contexts must be identical. This does not always mean that criteria and practices in the primary and target contexts must exactly match to guarantee transferability.

Since transferability is dependent on the conditions of each context, it is helpful to compare the primary and target contexts. This requires information from both the original context and the target context. In order to (1) determine whether and how (under what circumstances) the intervention is appropriate for improving the health of the target population, and (2) plan the transfer process systematically, it is imperative to identify the differences and similarities between the two contexts.^59^

### Study participants

The researcher aims to recruit a diverse group of stakeholders, representing a range of roles including health promotion professionals, programme leads, service providers, policymakers and researchers to explore how to transfer the SB intervention into real-world practice. We aimed to focus on stakeholders to assess the intervention’s wider transferability in practical settings. These stakeholders can offer important perspectives on the resource-related, logistical and policy-related aspects of executing an intervention into practice in various contexts. Their consultation was justified by the need to determine possible obstacles and enablers to scaling the intervention and to assess its adaptability and sustainability in various systemic and organisational contexts. Given the limited time and resources constraints, we were unable to include OAs in the transferability process of the intervention though we have conducted though we have carried out qualitative interviews to assess their acceptability, usability and feasibility in study 2. However, for the intervention’s transferability in a practical setting, we intend to conduct interviews with OAs in a larger effectiveness trial to find out the perception of OAs regarding transferability.

To recruit participants, key stakeholders will be selected who are involved in the implementation of interventions to enhance OAs’ activity and/or health and well-being.

#### Inclusion criteria

- Individuals who are involved in agenda setting, fund surveillance/monitoring and high-level support and advocacy for OAs’ health and well-being, for example, PA participation.
- Those who are involved in the provision of funding to develop, run and scale up interventions for increasing OAs’ health and well-being through PA programmes or fall prevention programmes.
- Those who oversee the running of the intervention. They may not directly engage with the participants but have knowledge of the development of OAs’ well-being, activity and SB interventions and all the components included.
- Those involved with the delivery of an intervention and engage face-to-face with participants. This can include the following personnel: Sports Development Officer, Safety Manager in fall prevention, Ageing Well Manager, Club/Group Coach, External Contractor, Health and Fitness Instructor.
- Individuals involved in research elements of the intervention for OAs’ health and well-being, that is, PA promotion or SB reduction, including pilot testing, feasibility trials, efficacy and/or effectiveness testing and evaluation.

#### Exclusion criteria

Participants will be excluded from the study if they are working with other groups in the population (eg, children).

### Study recruitment and setting

Regional coordinators and subcoordinators of key organisations in the city and county of Swansea will be contacted to enable stakeholders’ recruitment. Stakeholders they identify who are involved in planning, designing and implementing projects to support and enhance OAs’ activity or health and well-being will be contacted. This approach will help us to reach various stakeholders who work directly or indirectly with OAs. Recruitment letters, posters/leaflets will be used as an enrolment technique. Furthermore, we will advertise across the university using the intranet and work alongside the city and county of Swansea to recruit using their PA, play, health and community networks. Exploring stakeholders’ perspectives, as this study does, will help us to accommodate local needs when translating the intervention to increase activity and reduce SB in OAs from varied ethnic backgrounds in future effectiveness trials.

First, the lead researcher will explain the study and provide important study information to stakeholders. The researcher will contact interested participants via email or phone. Those who agree to take part will receive a participant information sheet. The lead researcher will explain the study process and procedures and answer any questions ensuring the stakeholder is fully informed of the nature of the study and how taking part will affect them. Stakeholders will also be informed that they can withdraw from the study at any time without facing any negative consequences. Additionally, they will be informed to get in touch with the study team if they desire to no longer participate in the research. Providing the stakeholders are happy to proceed, they will be given a consent form to sign prior to the interview. For any queries or issues, participants will be able to contact the primary researcher

### Patient and public involvement

There is no patient or public involvement in setting the research agenda.

### Data collection

A semi-structured interview guide was prepared to carry out IDIs (online supplemental file 2; Interview tool guide), based on the PIET-T.^60^ The PIET-T model can help with decision-making in this situation as a theoretical conceptual model for assessing the transferability of health interventions. Four key elements are necessary for this transferability: the population, the intervention, the environment in the primary and target contexts and the transference of the intervention.^60^

Participants will be informed that taking part in the research is voluntary, and they have the right to withdraw at any point. A unique ID number will be assigned to each participant and used for the duration of the study. Background information that includes age, gender, education and occupation will be gathered for descriptive purposes. The researcher will discuss the findings of the 12-week intervention to explain the intervention and then conduct IDIs with the participants to learn about their views on the transferability of the above-mentioned intervention in their context. IDIs work especially well for examining participants’ subjective feelings and perceptions in situations where a thorough understanding of the situation and context-specific information is crucial.^61^ Considering the complexity of evaluating the transferability of interventions, this qualitative method provides a solid basis for extracting comprehensive and significant information from stakeholders.^61^

The IDIs will explore various features including, but not restricted to: how the intervention aligns with policies, programmes and delivery methods; how the organisation (or other recommended organisations) might contribute to funding, approving or carrying out the programme in a possible real-world setting; (4) the feasibility of, and facilitators and barriers to, the intervention programme being transferred, and, (5) the recommended changes or modifications to enhance the uptake of the intervention in existing and potential initiatives that are delivered in the contexts in which the stakeholders work with OAs’ real world. The primary researcher (NAAM) will be the interviewer; she is trained and has expertise in qualitative research and has a good level of experience and expertise in conducting interviews. The mode by which the data will be collected will be based on the preference of the participants, that is, either face-to-face or online via Zoom/Teams. Both in-person or online interview options (via Teams or Zoom) will be offered in order to satisfy the demands and preferences of the stakeholders. This method assures inclusivity and flexibility, allowing participants to select the mode that best fits their particular situation. In-person interviews are beneficial for developing rapport, building trust and observing non-verbal cues—all of which are crucial in delicate or in-depth conversations. However, for stakeholders who could encounter logistical challenges including geographical distance, lack of time and other commitments, online interviews offer accessibility and feasibility.^62^ Providing both options enables a wider range of involvement and could result in more thorough and varied stakeholder perspectives. Moreover, both online and in-person interviews are effective, according to recent studies. Online interviews have been demonstrated to produce data of equivalent quality to in-person interviews, with the added advantages of greater accessibility and less participant burden.^63^ Hence, the hybrid approach adopted here is in line with contemporary qualitative research approaches that prioritise adaptability and participant-centric data collecting.

We will closely monitor the data collection process in accordance with best practices for qualitative research, initiating data analysis concurrent with data collection and determining whether any new themes or insights are still emerging from the interviews as the interviews progress. We will confirm saturation within categories that occur as substantial in the analysis process. This will be achieved when we observe that further interviews are no longer yielding new information to the study topics.^64^ Each interview will last approximately 30–40 min and will be audio-recorded.

### The 12-week intervention

The intervention on which interviews will be based is a multicomponent home-based programme to improve activity and minimise SB in OAs, which has been tested for feasibility and acceptability. The intervention, supported by the habit-based model and the social ecological model^19^ includes: (1) motivation for OAs to optimise their home space for activity (environmental level), (2) provision of individualised one-to-one health coaching session and pamphlet to increase awareness of SB and PA and their impact on health (individual level) and, (3) WATs and reminder messages to provide a prompt or cue to replace extended sitting with standing, stepping or light activity within their home setting, modifying their behaviour (individual level).

### Data analysis

The primary researcher (NAAM) will transcribe data from recorded interviews. To help confirm and cross-check the findings, the interviewer (NAAM) will collect field notes, which will be reviewed alongside the interview transcripts. NAAM will conduct reflexive thematic analysis using the steps outlined by Braun and Clarke.^65^ Reading interview notes and transcripts multiple times will be the first step in becoming familiar with the data (Step 1). The PIET-T model’s principles will direct the initial deductive coding process.^65^ Inductive coding will be used to create codes based on the data when it does not match the coding framework. Step 3 of the process will involve searching for themes and groupings associated with subcategories in order to create meaningful categories that will follow. Following this, themes will be examined (Step 4) and grouped according to the PIET-T model’s components (such as, intervention-PIET-T model) (Step 5). The final step will be writing up the findings (Step 6). Using a designated ID, anonymised written participant quotes will be used to provide a clear overview of the main themes. The qualitative data analysis programme NVivo V.12.0 will be used to organise the data in these steps. JH, the second author, will likewise code two transcripts at the beginning of Step 2. As a result, JH and NAAM will jointly review and assign the final themes.

### Ethics and dissemination

Stakeholders will be required to provide written informed consent prior to initiation of the study. Ethical approval for this study has been obtained from the College of Engineering Research Ethics Committee (320249732903), Swansea University. The study’s results will be shared with the scientific community through a peer-reviewed journal publication and with study participants through seminars and workshops.

## Discussion

This study will further add to the evidence base of investigations into perceptions of a diverse group of stakeholders as part of the intervention transferability phase of the research cycle.^40 66^ Importantly, this research has focused mostly on the USA and the current study will offer a perspective from UK stakeholders. This is a crucial step in facilitating intervention transferability to ensure programmes are prepared for implementation in real-world settings. The study’s findings will direct any necessary modifications and/or alterations in the intervention to improve its feasibility for real-world scale-up. Exploring stakeholders’ perspectives, as this study does, will help us to accommodate local needs when translating the intervention to increase activity and reduce SB in OAs from varied ethnic backgrounds in future effectiveness trials.

OAs from minority ethnic groups are harder to reach, less likely to participate in PA programmes and more prone to drop out from PA programmes^18^ due to numerous barriers for ethnically diverse OAs, including financial difficulties, social obligations, a lack of confidence, language hurdles, a lack of resources and restrictions based on religion and culture.^20^ Therefore, promoting PA among OAs, and in particular, OAs from diverse ethnic communities, should be a global priority as it enables active ageing and slows disability and disease progression.^19^ Since the suggested adaptations from stakeholders will be aimed at reaching an ethnically diverse population, this will undoubtedly help to increase equity of experience across different ethnic groups of OAs. Additionally, this will increase awareness of the promotion of PA among organisations. The finding of the study emphasise the support of stakeholders to facilitate the transition of research (or innovation) to real-world practice.^41^ Hence, the process of transferability must involve reciprocal learning between researchers and stakeholders; it cannot be one way. Moving forward, we intend to incorporate insights from stakeholders in implementing the intervention to test its effectiveness. Beyond the confines of research settings, this is a significant consideration for public health professionals to integrate PA (or health in general) initiatives into action within the community.

Within our time and resource constraints, we are unable to include OAs in exploring the transferability process of the intervention. However, we intend to conduct interviews with OAs in a larger effectiveness trial to find out how older individuals themselves feel about the intervention’s acceptability, usability and transferability into existing and potential initiatives delivered in real-world conditions. Through this interaction, we will be able to address any knowledge gaps on the target population’s requirements and preferences and improve the intervention further. In addition, this will enable us to triangulate the viewpoints of the OAs and the stakeholders, enabling us to ensure that the intervention is not only practical and acceptable from the perspective of the stakeholder but also in line with the factors that affect OAs’ participation in the intervention at home.

## Supplementary material

10.1136/bmjopen-2024-091049 online supplemental file 1

10.1136/bmjopen-2024-091049 online supplemental file 2
