# Supplementary material for: How to improve the transferability of a 12-week home-space sedentary behaviour intervention for ethnically diverse older adults: a qualitative study protocol of key stakeholder perspectives
Source: BMJ Open. 2025 Apr 17;15(4):e091049. doi: 10.1136/bmjopen-2024-091049 (PMC12007024; doi:10.1136/bmjopen-2024-091049)
Supplement: online supplemental file 2 [file bmjopen-15-4-s002.docx]

**Topic Sample Question**

**General questions:**

1. Have you ever worked in planning or organizing or implementing any program/intervention/project for older individuals’ well-being?
2. From your point of view, which factors contribute to a successful implementation of an intervention/program?
3. From your point of view, which factors hinder/ inhibit/ slow down the implementation of interventions?

Prompts: Prompts: (a. Training b. Staff expertise c. Involve community organization d. Stakeholders involved in the program implementation e. Monitoring of the intervention f. Tailor to target population need g. Culture-specific intervention for target population h. Intervention component easy to follow and understand i. Intervention accessible to participants j. Time issues affecting the implementation of the intervention k. Proper funding l. Process evaluation conducted m.Transferability of the intervention (integration into existing programs)

**Transferability of the feasibility intervention:**

1. Was the 12-week intervention program tailored to or meeting the needs of:

a.) the target population (e.g., socio-cultural background, gender, participants’ needs),

b.) local conditions or the setting,

c.) expertise of the staff responsible for implementing the intervention.

2. From your point of view, was the intervention easy to follow? Were the components easy to understand? If yes, how did the simplicity of the intervention facilitate transferability? If not, please elaborate how a lack of simplicity may have affected transferability?”

3. Our 12-week intervention included older people living in the community but not from healthcare services/ or in care homes. Do you think older people from service delivering/care homes should target for this intervention too?

4. Can intervention integrate into existing institutional programs, or any program offered by you? And what modifications needed in the intervention/(would it be needed)?

5.What support structures do you need to scale up/transfer this intervention? (Prompt: funding call/source, training, policy changes, research evidence base, etc) ?

7.From your point of view, what made this above-mentioned intervention sustainable? Which strategies were used to promote long-term transfer of the intervention?

8.What can you see would facilitate transferring this type of intervention at a state or national level?

9. What can you predict might be challenging if transferring this type of intervention at a state or national level? Why?

10. What factors lead to a trusting relationship with the participants that helps in the transferability of these interventions?

***We are just about finished: is there anything else you would like to add that might help us improve the transferability of interventions.***
