# Supplementary material for: How to improve the transferability of a 12-week home-space sedentary behaviour intervention for ethnically diverse older adults: a qualitative study protocol of key stakeholder perspectives
Source: BMJ Open. 2025 Apr 17;15(4):e091049. doi: 10.1136/bmjopen-2024-091049 (PMC12007024; doi:10.1136/bmjopen-2024-091049)
Supplement: Supplementary file 3 [file bmjopen-2024-091049.reviewer_comments.pdf]

PEER REVIEW HISTORY

BMJ Open publishes all reviews undertaken for accepted manuscripts. Reviewers are asked to complete a checklist review form and are provided with free text boxes to elaborate on their assessment. These free text comments are reproduced below.

ARTICLE DETAILS

Title (Provisional)

How to improve the transferability of a 12-week home-space sedentary behaviour intervention for ethnically diverse older adults: A qualitative study protocol of key stakeholder perspectives.

Authors

Meghani (NAAM), Naureen Akber Ali; Hudson (JH), Joanne; Stratton (GS), Gareth; Mullins, Jane

---

VERSION 1 - REVIEW

Reviewer 1

Name Teh, Ruth

Affiliation University of Auckland School of Population Health, Dept General Practice and Primary Health Care

Date 11-Nov-2024

COI None

Thank you for the opportunity to review this manuscript. Please see the attached feedback to enhance the clarity of the manuscript.

The reviewer provided a marked copy with additional comments. Please contact the publisher for full details.

Reviewer 2

Name Tong, Catherine

Affiliation University of Waterloo

Date 13-Jan-2025

COI None

Thank you for the opportunity to review this work, and for the team's efforts in advancing PA and reducing SB amongst older adults. This is important work. Below I raise several queries, and suggest many spots where additional information and/or citations would strengthen the

protocol as written. In its present form, the title does not match the written document (specifically the reference to ethnically diverse older adults).

Title and abstract:

- While the title references “ethnically diverse older adults”, the abstract does not discuss ethnically diverse older adults, which seems like an oversight or error.

- Given the international readership, I would also suggest identifying the setting (country/region) for the planned data collection in the abstract

Introduction:

- Given that the title references “ethnically diverse” older adults, I was expecting to see a section in the introduction that shows that some (but not all) ethnically diverse older adults, including those from migrant or minoritized or language-minority communities are generally less physically active than their local, native- born, non-racialized and/or English-speaking peers.

Methods:

- The authors completed interviews with diverse stakeholders, including those “representing a range of roles including health promotion professionals, programme leads, service providers, policymakers, and researchers to explore how to transfer the intervention into real-world practice.” Very few in the gerontological research community, or the implementation science community, would accept an exploration of the transferability of an intervention for older adults, without meaningful engagement with older adults. If that is not part of this planned/completed data collection, could this be a future/second phase of information gathering, prior to implementation? If that is not part of the planned or funded research project, this could be noted as a suggested or planned next step in the discussion. Please provide a justification here, or in the introduction, as to why these sorts of stakeholders are the most appropriate people to provide information to answer your research questions and/or help set the research agenda, rather than older adults themselves (particularly given the home-based nature of the intervention).

- Regarding the sentence “are not able to cooperate with the research team for the full duration of the project”. This is a problematic statement, chiefly the term “cooperate”; it also vague. Why does a participant need to ‘cooperate’ for the duration of the project, once their interview is complete? Alternatively, you could provide information regarding whether a participant can withdraw from the study, at what point, and what is the process to do so. That would probably be clearer.

- If transcription has not yet occurred, would highly recommend using support tools for the transcription (e.g. otter.ai). While imperfect, they can save a lot of time.

- Please provide a reference regarding saturation, and how will the research team know /determine if “saturation” has been reached? I would recommend consulting this paper:

Morse, J. M. (2015). Data were saturated... Qualitative health research, 25(5), 587-588.

And the concept of Information Power could be considered as an alternative, see:

Malterud, K., Siersma, V. D., & Guassora, A. D. (2016). Sample size in qualitative interview studies: guided by information power. Qualitative health research, 26(13), 1753-1760.

- Please provide references to support your data analysis plans

- Regarding this sentence: "The coding, development of categories, and reflexive thematic analysis will be carried out by two separate researchers", it would be helpful to provide some additional detail regarding your approach to team-based analysis (and references). As is, this sentence could be interpreted as the researchers doing their analyses completely separately, which would then be difficult to reconcile, or it could be interpreted as "the researchers will engage in a combination of independent line-by-line coding and team-based analysis meetings to identify the thematic framework, define codes, and chart the data" (here you can also refer to the steps typically used in thematic analysis, see the many articles by or based on Braun & Clarke's work).

- Can you please add some information on if/how the theoretical model (SEM) mentioned in the introduction impacted either the planned data collection (e.g. the interview guide questions) and/or the planned analysis.

Discussion:

- Regarding this statement: "This study will contribute to a limited evidence base of investigations into perceptions of a diverse group of stakeholders as part of the intervention transferability phase of the research cycle." Please note that there is a sizeable body of literature, most of which is from the United States, on transferring and adapting physical activity interventions for diverse communities, in working with diverse stakeholders. That work should be cited, rather than stating here that there is a limited evidence base.

VERSION 1 - AUTHOR RESPONSE

**Reviewer 1 Comments to Author:**	**Responses**:
Reviewer: 1<br>Dr. Ruth Teh, University of Auckland School of Population Health<br>Comments to the Author:<br>Thank you for the opportunity to review this manuscript. Please see the attached feedback to enhance the clarity of the manuscript.	Thank you so much for sharing your expertise with us; we appreciate the time you have taken to scrutinise the paper.
potentially increasing the risk of chronic ailments- Please be clearer/more specific what do you means by chronic ailments.	The word chronic ailment has been changed to chronic diseases. Changes are done on page 4, line no. 39.

Real-world contexts - what does it mean by this, still home space or in the community? more elaboration would be helpful.	Thank you so much for your feedback. Changes are done on page 5, line number 5-7.
This framework describes how different levels of factors—including intrapersonal, interpersonal, organisational, environmental and policy—affect the individual- this sentence is ambiguous. who is 'individual' referring to?	Thank you so much for your feedback. This sentence has been removed. We have revised the model. The model is summarized in the method section. Changes are done on page 6-line no. 22 -43 and on page 7-line no. 1 -13.
To gain this knowledge about stakeholders' perspectives regarding the transferability of a 12-week intervention programme for sedentary and physically inactive OAs, qualitative methods will be employed-It is unclear if the stakeholders were involved in the design/development of the 12-week intervention study. Please clarify:	Thank you so much for your feedback. We have revised the aim. Changes are done on 5, line no. 44-45 and page 6-line no. 1-4.<br><br>In this regard, the 12-week intervention that was designed by the researcher and implemented on the ethnically diverse older adults will be presented to stakeholders to gain knowledge about their perceptions to address intervention transferability. Therefore, the current study aims to explore stakeholders' perceptions on the design and delivery of a home-based sedentary behaviour intervention implemented with ethnically diverse sedentary OAs to inform transferability (future implementation and scaling up).
Methods:<br>Study Design<br>*Before the interview the primary researcher will explain the intervention and discuss the findings of the feasibility study* - The study aim could be clearer. Please include the aim of the feasibility study, which is usually not power to examine the effectiveness of an intervention programme.<br>A clearer connection between this qualitative study and in the context of the feasibility study is desired.	Thank you so much for your feedback. We have revised the aim. Changes are done on page 6-line no. 1-4 and line no. 9-21.<br><br>Thank you so much for your feedback. We have revised the design part in method section. Before the interview the primary researcher will explain the intervention that has been assessed in a previous feasibility study (57) where sedentary OAs were asked to wear an activity tracker to remind them to cut down their sitting time (by standing up and moving more), attended a brief health coaching session, and were provided with a pamphlet and weekly reminder messages to decrease their sedentary behaviour and enhance their physical activity within the home. The current formative research builds on this feasibility study by enabling stakeholder engagement to involve end users' in planning for the intervention's transferability and scalability, shown to be significant in fostering intervention uptake (58). This will help us to understand the practical issues or possible challenges of delivering this intervention at larger scale within current policy and service contexts. The interview will then focus on perceptions of the transferability of the intervention in the stakeholder context. The consolidated checklist, Supplementary file 1, contains guidelines for qualitative research that will be followed in reporting this study.
please be more specific, i.e. PA intervention or any intervention	Thank you so much for your feedback. We have revised the sentence. The researcher aims to recruit a diverse group of stakeholders, representing a range of roles including health

	promotion professionals, programme leads, service providers, policymakers, and researchers to explore how to transfer the sedentary behaviour intervention into real-world practice.<br>Changes are done on page no. 7, line no. 17.
Please rephrase: The primary researcher will be contacted if participants have any queries or issues with the research.	Thank you so much for your feedback. We have revised the sentence. For any queries or issues participants can contact the primary researcher. Changes are done on page no. 8, line no. 26-27
**Data collection:**<br><br>please clarify, is it a focus group or one-on-one interview?<br><br>current initiatives ambiguous. please clarify<br><br>rephrase: enhance uptake in the real world (with evidence on the effectiveness of an intervention)<br><br>please clarify the research experience of the interviewer, are the trained and experienced interviewer?<br><br>Q. please elaborate and justify the chosen interview mode supported with refs. (how to response I have included both)	**Data collection:**<br><br>Thank you so much for your feedback. We have revised the section. Changes are done on page no. 8, line no. 31-43 & page 9 1-33.<br>A semi-structured interview guide was prepared to carry out IDIs based on the Population-Intervention-Environment-Transfer Model of Transferability (PIET-T) (60). The PIET-T model can help with decision-making in this situation as a theoretical conceptual model for assessing the transferability of health interventions. Four key elements are necessary for this transferability: the population, the intervention, the environment in the primary and target contexts, and the transference of the intervention (60).<br>Participants will be informed that taking part in the research is voluntary, and they have the right to withdraw at any point. A unique ID number will be assigned to each participant and used for the duration of the study. Background information that includes age, gender, education, and occupation will be gathered for descriptive purposes. The researcher will discuss the findings of the 12-week intervention to explain the intervention and then conduct IDIs with the participants to learn about their views on the transferability of the above-mentioned intervention in their context. IDIs work especially well for examining participants' subjective feelings and perceptions in situations where a thorough understanding of the situation and context-specific information is crucial (61). Considering the complexity of evaluating the transferability of interventions, this qualitative method provides a solid basis for extracting comprehensive and significant information from stakeholders (61).<br><br>The IDIs will explore various features including, but not restricted to: How the intervention aligns with policies, programmes, and delivery methods; How the organisation (or other recommended organisations) might contribute to funding, approving, or carrying out the programme in a possible real-world setting; 4) The feasibility of, and facilitators and barriers to, the intervention programme being transferred, and, 5) The recommended changes or modifications to enhance the uptake of the intervention in existing and potential initiatives that are delivered in the

	contexts in which the stakeholders work with OAs' real world. The primary researcher (NAAM) will be the interviewer; she is trained and has expertise in qualitative research and has a good level of experience and expertise in conducting interviews. The mode by which the data will be collected will be based on the preference of the participants i.e., either face-to-face or online via Zoom©/Teams©. Both in-person or online interview options (via Teams© or Zoom©) will be offered in order to satisfy the demands and preferences of the stakeholders. This method assures inclusivity and flexibility, allowing participants to select the mode that best fits their particular situation. In-person interviews are beneficial for developing rapport, building trust, and observing nonverbal cues—all of which are crucial in delicate or in-depth conversations. However, for stakeholders who could encounter logistical challenges including geographic distance, lack of time, and other commitments, online interviews offer accessibility and feasibility (62). Providing both options enable a wider range of involvement and could result in more thorough and varied stakeholder perspectives. Moreover, both online and in-person interviews are effective, according to recent studies. Online interviews have been demonstrated to produce data of equivalent quality to in-person interviews, with the added advantages of greater accessibility and less participant burden (63). Hence the hybrid approach adopted here is in line with contemporary qualitative research approaches that prioritise adaptability and participant-centric data collecting.<br>We will closely monitor the data collection process in accordance with best practices for qualitative research, initiating data analysis concurrent with data collection, and determine whether any new themes or insights are still emerging from the interviews as the interviews progress. We will confirm saturation within categories that occur as substantial in the analysis process. This will be achieved when we observe that further interviews are no longer yielding new information to the study topics (64). Each interview will last approximately 30-40 min and will be audio-recorded.
Data analysis<br>***The primary researcher will transcribe the recorded interview data. NVivo V.12 Plus software will be used to import, organise, and analyse data for analysis, and reflexive thematic analysis will be performed***. Add Reference:<br><br>How many coders. Please describe how themes are check and verify.	Thank you so much for your feedback. We have revised entire analysis part. Changes are done on page no. 9, line no. 44-45 & page 10, line no. 1-12.<br><br>**Data analysis:**<br>The primary researcher (NAAM) will transcribe data from recorded interviews. To help confirm and cross-check the findings, the interviewer (NAAM) will collect field notes, which will be reviewed alongside the interview transcripts. NAAM will conduct reflexive theme analysis using the steps

	outlined by Braun and Clarke (63). Reading interview notes and transcripts multiple times will be the first step in getting to know the data (Step 1). The PIET-T model's principles will direct the initial deductive coding process (63). Inductive coding will be used to create code based on the data when it does not match the coding framework. Step 3 of the process is searching for themes and groupings associated with subcategories (like intervention convenience and simple in order to create meaningful categories that will follow. After that, themes will be examined (Step 4) and grouped according to the PIET-T model's components (like Intervention-PIET-T model) (Step 5). The final step will be written up the findings (Step 6). Using a designated ID, anonymized written participant quotes will be used to provide a clear overview of the main themes. The qualitative data analysis program NVivo version 12.0 will be used to organize the data in these steps. JH, the second author, likewise coded two transcripts at the beginning of Step 2. As a result, JH and NAAM will jointly review and assign the final themes.
Discussion:<br><br>need to be clearer in the method section on achieving this goal. It is unclear at the moment. It is also desirable to include commentary on the ethnic diversity in study area/region	Thank you so much for your feedback. We have revised entire discussion part. Changes are done on page 10, line no. 20-46 & page 11, line no. 1-8.

Reviewer 2 Comments to Author	Responses
Dr. Catherine Tong, University of Waterloo<br>Comments to the Author:<br>Thank you for the opportunity to review this work, and for the team's efforts in advancing PA and reducing SB amongst older adults. This is important work. Below I raise several queries, and suggest many spots where additional information and/or citations would strengthen the protocol as written.	Thank you so much for sharing your expertise with us; we appreciate the time you have taken to scrutinise and improve our protocol.<br><br>Thank you so much for your feedback. We have revised the title.
In its present form, the title does not match the written document (specifically the reference to ethnically diverse older adults).	Thank you so much for your feedback. We have revised the title.<br><br>**Study Title:**<br>How to improve transferability of a 12-week home-space sedentary behaviour intervention for ethnically diverse older adults: A qualitative study protocol of key stakeholder perspectives
Title and abstract:<br>- While the title references “ethnically diverse older adults”, the abstract does not discuss ethnically diverse older adults, which seems like	Thank you so much for your feedback. We have revised abstract part. Changes are done on page 2, line no. 4 -23

an oversight or error.<br>- Given the international readership, I would also suggest identifying the setting (country/region) for the planned data collection in the abstract	
Introduction:<br>- Given that the title references “ethnically diverse” older adults, I was expecting to see a section in the introduction that shows that some (but not all) ethnically diverse older adults, including those from migrant or minoritized or language-minority communities are generally less physically active than their local, native- born, non-racialized and/or English-speaking peers.	Thank you so much for your feedback. We have incorporated feedback. Changes are done on page 4, line no. 11-46 and page 5, line no. 1-7.
Methods:<br>- The authors completed interviews with diverse stakeholders, including those “representing a range of roles including health promotion professionals, programme leads, service providers, policymakers, and researchers to explore how to transfer the intervention into real-world practice.” Very few in the gerontological research community, or the implementation science community, would accept an exploration of the transferability of an intervention for older adults, without meaningful engagement with older adults. If that is not part of this planned/completed data collection, could this be a future/second phase of information gathering, prior to implementation? If that is not part of the planned or funded research project, this could be noted as a suggested or planned next step in the discussion. Please provide a justification here, or in the introduction, as to why these sorts of stakeholders are the most appropriate people to provide information to answer your research questions and/or help set the research agenda, rather than older adults themselves (particularly given the home-based nature of the intervention).	Thank you for your insightful comments. We recognise the value of actively interacting with elderly people when investigating the transferability of interventions meant to enhance their health outcomes, especially when it comes to a home-based intervention. We aimed to focus on stakeholders to assess the intervention's wider acceptability and transferability in practical settings. These stakeholders can offer important perspectives on the resource-related, logistical, and policy-related aspects of executing an intervention into practice in various contexts. Their consultation was justified by the need to determine possible obstacles and enablers to scaling the intervention and to assess its adaptability and sustainability in various systemic and organisational contexts. We do, however, agree with the feedback that direct input from older adults should be considered when evaluating transferability. Although older individuals were not directly included in the transferability evaluation part of our study, we concur that their viewpoints are essential to comprehend how the intervention might be embraced, incorporated, and maintained in their daily life. We therefore acknowledge this as a crucial next stage in the process of larger effectiveness trial. Given the limited time and resources constraints we were unable to include older adults in the transferability process of the intervention. However, we intend to conduct interviews with older adults in lager effectiveness trial to find out how older individuals themselves feel about the intervention's acceptability, usability, and transferability in practical setting. Through this interaction, we will be able to address any knowledge gaps on the target population's requirements and preferences and improve the intervention even more. In addition, this will enable us to triangulate the viewpoints of the older adults and the stakeholders, providing that the intervention is not only practical and acceptable from the perspective of the service provider but also in line with the practical and compelling factors that affect older adults' participation in the intervention at home.

- Regarding the sentence “are not able to cooperate with the research team for the full duration of the project”. This is a problematic statement, chiefly the term “cooperate”; it also vague. Why does a participant need to ‘cooperate’ for the duration of the project, once their interview is complete? Alternatively, you could provide information regarding whether a participant can withdraw from the study, at what point, and what is the process to do so. That would probably be clearer.	Thank you so much for raising this point, the term co-operate has been removed. Changes are done on page 8, line no. 22-27.<br><br>**Response:** We appreciate your insightful comments about the phrase "cooperate with the research team." We are aware that the word "cooperate" could be interpreted as ambiguous and troublesome. Therefore, we have removed this phrase. In addition, we think that it's critical to inform participants that they withdraw anytime from research, therefore we have added phrase.<br>Stakeholders will also be informed that they can withdraw the study at any time without facing any negative consequences. Additionally, they will inform to get in touch with the study team if they desire to no longer participate in research. Providing the stakeholders are happy to proceed, they will be given a consent form to sign prior to the interview. For any queries or issues participants can contact the primary researcher
- If transcription has not yet occurred, would highly recommend using support tools for the transcription (e.g. otter.ai). While imperfect, they can save a lot of time.	Thank you so much for raising this point, this is a very excellent idea that can be used for future studies.
- Please provide a reference regarding saturation, and how will the research team know /determine if “saturation” has been reached? I would recommend consulting this paper:<br><br>Morse, J. M. (2015). Data were saturated... Qualitative health research, 25(5), 587-588.<br><br>And the concept of Information Power could be considered as an alternative, see:<br><br>Malterud, K., Siersma, V. D., & Guassora, A. D. (2016). Sample size in qualitative interview studies: guided by information power. Qualitative health research, 26(13), 1753-1760	Thank you so much for raising this point, the term co-operate has been removed. Changes are done on page 9, line no. 28-33<br>We will closely monitor the data collection process in accordance with best practices for qualitative research and determine whether any new themes or insights are still emerging from the interviews. We will confirm saturation within categories that occur as substantial in the analysis process. The saturation will be achieved when we observe that further interviews are no longer yielding new information to the study topics (63). In addition, it is witnessed that the sample holds greater information that is appropriate for the study to direct sufficient sample for qualitative studies (64).
- Please provide references to support your data analysis plans<br><br><br>- Regarding this sentence: “The coding, development of categories, and reflexive thematic analysis will be carried out by two separate researchers”, it would be helpful to provide some additional detail regarding your approach to team-based analysis (and	Thank you so much for this point. The analysis plan has modified. Changes are done on page 9, line number 44-45 and page 10, line no. 1-12.<br><br><br>The primary researcher (NAAM) will transcribe data from recorded interviews. To help confirm and cross-check the findings, the interviewer (NAAM) will collect field notes, which will be reviewed alongside the interview transcripts. NAAM will conduct reflexive theme analysis using the steps outlined by Braun and Clarke (65). Reading interview notes and transcripts multiple times will be the

references). As is, this sentence could be interpreted as the researchers doing their analyses completely separately, which would then be difficult to reconcile, or it could be interpreted as “the researchers will engage in a combination of independent line-by-line coding and team-based analysis meetings to identify the thematic framework, define codes, and chart the data” (here you can also refer to the steps typically used in thematic analysis, see the many articles by or based on Braun & Clarke’s work).	first step in getting to know the data (Step 1). The PIET-T model's principles will direct the initial deductive coding process (65). Inductive coding will be used to create code based on the data when it does not match the coding framework. Step 3 of the process is searching for themes and groupings associated with subcategories (like intervention convenience and simple in order to create meaningful categories that will follow. After that, themes will be examined (Step 4) and grouped according to the PIET-T model's components (like Intervention-PIET-T model) (Step 5). The final step will be written up the findings (Step 6). Using a designated ID, anonymized written participant quotes will be used to provide a clear overview of the main themes. The qualitative data analysis program NVivo version 12.0 will be used to organize the data in these steps. JH, the second author, likewise coded two transcripts at the beginning of Step 2. As a result, JH and NAAM will jointly review and assign the final themes
- Can you please add some information on if/how the theoretical model (SEM) mentioned in the introduction impacted either the planned data collection (e.g. the interview guide questions) and/or the planned analysis.	Thank you so much for your feedback. We have used PIET-T model for transferability Population-Intervention-Environment-Transfer Model of Transferability (PIET-T)<br>Changes are done on page 6, line number 22-43 and page 7, line no. 1-12.
Discussion:<br>- Regarding this statement: “This study will contribute to a limited evidence base of investigations into perceptions of a diverse group of stakeholders as part of the intervention transferability phase of the research cycle.” Please note that there is a sizeable body of literature, most of which is from the United States, on transferring and adapting physical activity interventions for diverse communities, in working with diverse stakeholders. That work should be cited, rather than stating here that there is a limited evidence base.	Thank you so much for your feedback. Changes are done on page no. 10, line no. 21-47, page no. 11 line no .1 -10
